# Evaluation of the NG-Test Carba 5 for the clinical detection of carbapenemase-producing gram-negative bacteria

**DOI:** 10.3389/fmed.2025.1512345

**Published:** 2025-03-14

**Authors:** Hai-Feng Qin, Jin-Ke He, Xin Chen, Ke Jiang, Xiao-Yan Cai, Xiao-Ni Wu, Lei Ye, Hao-Kai Chen, Xu-Guang Guo, Yong Xia

**Affiliations:** ^1^Department of Clinical Laboratory Medicine, Guangdong Provincial Key Laboratory of Major Obstetric Diseases, Guangdong Provincial Clinical Research Center for Obstetrics and Gynecology, The Third Affiliated Hospital, Guangzhou Medical University, Guangzhou, China; ^2^Department of Clinical Medicine, The Third Clinical School of Guangzhou Medical University, Guangzhou, China; ^3^Department of Clinical Medicine, The Second Clinical College of Guangzhou Medical University, Guangzhou, China; ^4^Guangzhou Key Laboratory for Clinical Rapid Diagnosis and Early Warning of Infectious Diseases, King Med School of Laboratory Medicine, Guangzhou Medical University, Guangzhou, China

**Keywords:** NG-Test Carba 5, immunochromatographic assay, carbapenemase-producing, carbapenem-resistant gram-negative bacteria, clinical detection

## Abstract

**Background:**

Currently, the spread and prevalence of carbapenem-resistant gram-negative bacteria cause a worldwide health problem, significantly affecting patients’ prognosis. Therefore, reliable detection of carbapenemases is crucial for managing and controlling infections. Numerous investigations have shown that the innovative immunochromatographic assay NG-Test Carba 5 has great sensitivity and specificity for carbapenemase typing. This meta-analysis aimed to comprehensively assess the efficacy of the NG-Test Carba 5 in the clinical detection of carbapenemase-producing gram-negative bacteria.

**Methods:**

Previously published articles were systematically reviewed, relevant data were extracted, and the results were pooled and analyzed using Meta-DiSk 1.4 and Stata 12.0 software.

**Results:**

The sensitivity, specificity, positive LR value, and negative LR value for the identification of carbapenemase-type KPC, NDM, VIM, IMP, and OXA-48-like by immunochromatographic NG-Test Carba 5 using PCR as gold standard were 0.97 [95% CI (0.97, 0.98)], 0.99 [95% CI (0.99, 1.00)], 65.38 [95% CI (36.73, 116.39)], and 0.03 [95% CI (0.02, 0.05)], respectively, and the combined diagnostic odds ratio was 2,734.42 [95% CI (1,464.05, 5,107.12)]. The AUC of the SROC curve was 0.9976.

**Conclusion:**

In summary, the NG-Test Carba 5 is a clinical test that can identify and quickly detect five major carbapenemases, thus offering valuable insights for clinical decision-making and infection control.

## Introduction

The emergence and prevalence of carbapenem-resistant gram-negative bacteria (CR-GNB) are gradually becoming a threat to public health, with carbapenem-resistant Enterobacteriaceae (CRE), carbapenem-resistant *Pseudomonas aeruginosa*, and carbapenem-resistant *Acinetobacter baumannii* being classified as the highest-priority (i.e., critical) pathogens by the World Health Organization (WHO) (1). Infections caused by CRE usually result in high mortality and poor prognosis due to the ability of carbapenemases to hydrolyze all β-lactam antibiotics resulting in few antibiotics retaining activity against CRE (2). Therefore, without timely early treatment and infection control, patients will have an increased risk of developing mortality. And for specific high-volume areas, it will only increase the difficulty of treatment, prolong hospitalization, and aggravate the pressure of hospital infection prevention and control, which will continue to deteriorate week after week. Thus, it is clinically critical to diagnose carbapenemase infections as soon as possible. So, we need simple, rapid, effective and inexpensive techniques to screen infected patients for the presence of these pathogens.

Researchers systematically categorized the carbapenemases into four groups: A, B, C, and D. Group A included most of the KPC strains; group B included many VIM, IMP, and NDM strains with carbapenemase activity detected from gram-negative bacteria; group C included AmpC β-lactamase, but it can play a role in the hydrolysis of carbapenemase only under special circumstances (osmotic abnormality); and group D included the OXA-48 type of Enterobacteriaceae bacteria (1). In summary, under normal conditions, classes A, B, and D are able to exert carbapenemase activity, thereby achieving resistance.

The modified Carbapenem Inactivation Method combined with the EDTA Carbapenem Inactivation Method (mCIM/eCIM) recommended by the Clinical and Laboratory Standardization Institute (CLSI) guidelines has excellent precision in carbapenemase assays but at the expense of a lengthy turnaround time (TAT) (3). Currently, most of the tests for carbapenemases in clinics and laboratories are biochemical and molecular, including phenotypic analysis of carbapenemase activity, polymerase chain reaction (PCR) assays of carbapenemase genes, commercial microarray assays (1), whole-genome sequencing (WGS), and assay techniques such as Xpert Carba-R and NG-Test Carba 5. Compared with NG-Test Carba 5, conventional assay technologies still have significant drawbacks.

In addition, the NG-Test Carba 5 is a novel immunochromatographic assay evaluated for use in many studies (4–7) that qualitatively detects carbapenemases such as KPC, NDM, IMP, VIM, OXA-48, etc., through the particular binding of the antigen to monoclonal antibodies; detects carbapenemase typing, which requires only 20 min; and is characterized by ease of operation, quick detection speed, and other qualities. According to a study by Han et al. (2), the NG-Test Carba 5 performed well overall, with a sensitivity and specificity ranging from 95.3% to 100% and 97.3% to 100%, respectively, on colonies of bacteria and positive cultures of blood. The NG-Test Carba 5 was shown to be more efficient in terms of turnaround time, initial and ongoing expenses, and sensitivity in a study by Kanahashi et al. (4). The Xpert Carba-R assay was not as effective as the NG-Test Carba 5. For certain strains that test negative, there is a chance that the Carba 5 NG-Test will go undetected.

To conduct evidence-based research and provide a thorough assessment of the detection ability of carbapenemase-producing gram-negative bacteria (CP-GNB) by the NG-Test Carba 5, a meta-analysis was performed in this study. The research group searched PubMed for meta-analysis papers related to this topic, but there were no such papers. This is the first study to apply evidence-based medical evidence, such as the sensitivity and specificity of the NG-Test Carba 5 for the clinical detection of CP-GNB, and we hope that this study offers fresh perspectives on the rapid clinical detection of GNB resistance by the NG-Test Carba 5 and contributes to the development of the field.

It is worth mentioning that the novelty of this study compared with the existing researches is that the researchers designed three subgroups, namely, detection outcome (Group A), bacterial species (Group B) and bacterial isolation methods (Group C). For the first time, we provided a statistical reference for the bacterial isolation methods of NG-Test Carba 5 in clinical application, as well as a comprehensive analysis of the accuracy and differences between different subgroups, so that NG-Test Carba 5 can further improve its own detection performance to provide a certain evidence-based basis.

## Materials and methods

### Study design

A systematic evaluation of the accuracy of the NG-Test Carba 5 for diagnostic carbapenemase typing followed by a meta-analysis was performed.

### Electronic search

Researchers conducted searches in the following four databases: Web of Science, Embase, Cochrane Library, and Pubmed. Publications from May 2019 to January 2025 that contained the terms “NG-Test CARBA 5,” “Carbapenemase-Producing,” and “Gram Negative Bacteria” were gathered. Synonyms of Carbapenemase-Producing are Carbapenem-Resistant, KPC, NDM, IMP, VIM, OXA-48-like; synonyms of Gram Negative Bacteria are Enterobacterales, *Pseudomonas aeruginosa*, *Acinetobacter baumannii*. Then synonyms were linked using OR, and Carbapenemase-Producing and Gram Negative Bacteria were linked with OR before linking with NG-Test CARBA 5 using AND.

The six researchers were divided into three groups. The first group of researchers (Xiao-Yan Cai, Ke Jiang) screened the literature in the order of screening out duplicates, going through each title and abstract individually, and then reading the entire text in accordance with the predefined inclusion and exclusion criteria. The second group of researchers (Hai-Feng Qin, Xin Chen) extracted the data, evaluated the quality, and organized the summary of the included literature. Two people in each group performed the analysis independently, followed by data cross-approval. If the two groups of researchers disagreed on the data extraction results, a third group of researchers (Xiao-Ni Wu, Lei Ye) was introduced to negotiate and resolve the issue together, and this third group of researchers remained blinded to the articles under review.

### Inclusion and exclusion criteria

The inclusion criteria were as follows: (i) clinical specimens or standard bacteria diagnosed with carbapenemase-type KPC, NDM, VIM, IMP or OXA-48-like by the immunochromatographic method NG-Test Carba 5. (ii) The gold standard is PCR. (iii) Limited to the English literature. (iv) Information in the four grid tables can be obtained directly or through indirect calculations.

The exclusion criteria were as follows: (i) Duplicate studies, abstracts, conference abstracts, case reports, assessments, letters, or unrelated articles. (ii) Articles lacking a gold standard or unable to extract the four-grid table.

### Data extraction

The extracted content included the writer’s name, year of publication, strain distribution area, research methodology (prospective or retrospective), specimen source (clinical sample or standard bacteria), specimen type (rectal swabs or blood samples, etc.), specimen processing method (blood agar culture or others), gold standard, TP, FP, FN, TN, sensitivity, specificity, total number of specimens, test outcomes, bacterial species, and other related information, using the four-grid table as a standardized form of data extraction.

### Quality evaluation

The second group of researchers evaluated the included literature using a standardized quality evaluation form with QUADAS-2 criteria. The study QUADAS-2 quality criteria were provided by Review Manager 5.2 and consisted of four domains (Patient Selection, Indexed Detection, Process and Time, and Reference Standards) in eleven subitems for specific evaluation.

### Statistical analysis

The study quality was assessed using Review Manager version 5.3. The software Meta-DiSk 1.4 was used to conduct the pooled analysis. To evaluate the precision of the NG-Test Carba 5 for diagnostic carbapenemase typing, the combined parameters of the forest plot were calculated with a random-effects model. These calculations included the determination of sensitivity, specificity, positive likelihood ratio (PLR), negative likelihood ratio (NLR), diagnostic odds ratio (DOR), and 95% CIs. To ascertain whether a threshold effect was present, the threshold effect study computed Spearman’s correlation coefficient and examined the SROC curves. The area under the curve (AUC) was also estimated, and the AUC was not dependent on the diagnostic threshold, with the AUC of a good diagnostic test being close to 1. The I^2^ test assessed the heterogeneity of the qualifying studies. The heterogeneity between studies affects how the effects model is applied. If there was no significant heterogeneity in the included studies (I^2^ < 50%), the results were analyzed through a fixed-effects model; if there was considerable heterogeneity in the eligible studies (I^2^ = 50%), a random-effects model was applied for the meta-analysis. To assess publication bias, Stata 12.0 software was used to construct funnel plots.

## Results

### Selection and characterization of the included studies

**FIGURE 1:**
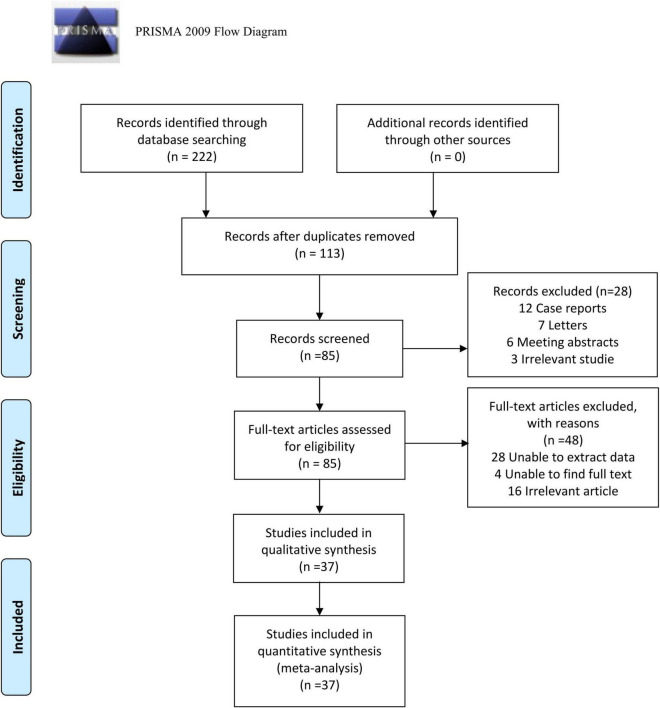
The procedure for identifying and choosing literature.

The procedure for identifying and choosing literature is illustrated in Figure 1. In accordance with the search plan, 222 publications were identified. After duplicates were eliminated, 113 publications remained. Twelve case reports, seven letters, six conference abstracts, and three irrelevant publications were excluded following an examination of the titles and abstracts. Upon reviewing the entire text, 28 publications from which data could not be extracted, four from which the full text could not be found, and 16 unrelated publications were further excluded. Finally, 37 articles that met the inclusion criteria were included (2–38), and their study data were extracted for meta-analysis (Table 1). The 37 investigations had 9,153 samples in total, and 37 sets of four-compartment table data were extracted.

**TABLE 1:** Characteristics of the included studies.

References	Country	Geographical distribution of strains	Study design	Source of specimens	Type of specimens	Bacterial isolates methods	Gold standard	Total	TP	FP	FN	TN	Correct rate %
Tartor et al. (31)	Egypt	Egypt	Prospective	Clinical isolates	Rectal swabs	MacConkey agar	PCR	20	16	0	4	0	0.8
Lee et al. (32)	China	China	Prospective	Clinical isolates	–	CPS agar and blood agar	WGS	30	2	0	0	28	1
Munguia-Ramos et al. (33)	Mexico	Mexico	Retrospective	Clinical isolates	Blood	Blood culture and artificially inoculated blood culture	PCR	32	14	0	1	17	0.97
Bianconi et al. (34)	Italy	Northern Italy	Retrospective	Clinical isolates	Ulcer\urine\recta blood\feces\wound\pus	Blood agar plate	MALDI-TOF	26	26	0	0	0	1
Gao et al. (35)	China	China	Prospective	Clinical strains	Sputum\urine\abdominal fluid\cerebrospinal fluid	blood agar	WGS	58	57	0	1	0	0.98
Lin et al. (36)	China	China	Retrospective	Clinical isolates	–	Sheep blood plates and tryptone soy broth	PCR	477	346	12	3	116	0.97
Wang et al. (37)	China	China	Retrospective	Clinical isolates	Sputum\bronchoal veolar lavage\urine\blood\bile	Single colony	WGS	2665	2575	0	90	0	0.97
Tarlton et al. (30)	United States	United States	Retrospective	Clinical isolates	–	–	WGS	11	6	0	0	5	1
Wang et al. (38)	China	China	Prospective	Clinical Samples	Rectal swabs	CHROMagar	PCR	200	96	0	4	100	0.98
Mendez-Sotelo et al. (8)	Mexico	Mexico	Prospective	Clinical isolates	–	Blood culture	PCR	84	69	0	5	10	0.94
Khoo et al. (6)	Singapore	Singapore	Prospective	A mix of surveillance rectal swabs and clinical samples	Rectal swabs	Chrom ID CARBA SMART Agar	WGS	235	140	0	4	91	0.98
Gu et al. (9)	China	China	Retrospective	Clinical isolates and hospital sewages	Clinical isolates and hospital sewages	Blood agar	WGS	207	201	0	6	0	0.97
Satoshi, 2022	Japan	Japan	Retrospective	Clinical isolates	Pharyngeal mucosa\fecal\urine samples\abdominal drains rectal vaginal swabs	Single colony	PCR	540	86	0	0	454	1
Saito et al. (5)	Japan	Japan	Retrospective	Environment (river water or hospital sewage) and clinical isolates	–	Blood culture	PCR and DNA sequencing	164	106	0	1	57	0.99
Zhang et al. (11)	China	China	Prospective	Clinical isolates	Sputum\blood\urine\specimens	Columbia blood agar plates	DNA sequencing	247	227	0	1	19	1
Huang et al. (12)	Taiwan, China	Taiwan, China	Retrospective	Laboratory isolate strains	Blood isolates	Blood agar	PCR	214	87	1	0	126	1
Josa et al. (7)	Colombia	Colombia	Prospective	Laboratory isolate strains	–	MacConkey agar	PCR	100	86	0	0	14	1
Comini et al. (13)	Italy	Italy	Prospective	Clinical isolates	Blood	Direct clinical blood testing	PCR	478	165	0	14	299	0.97
Stokes et al. (14)	Canada	Canada	Prospective	Clinical samples and seeded samples	–	Blood culture	PCR	65	27	0	3	35	0.95
Vasilakopoulou et al. (15)	Greece	Greece	Prospective	Clinical isolates	Rectal swabs	Direct clinical blood testing	PCR	20	20	0	0	0	1
Yoon et al. (16)	Korea	Korea	Both^1^	Clinical isolates	Rectal swabs\urine\body fluids\respiratory specimens\tissues	MacConkey agar	PCR	148	114	0	1	33	0.99
Zhu et al. (17)	China	China	Prospective	–	Respiratory\sterile fluid tissues\blood\rectal swab/stool urine	Blood agar	PCR	299	253	1	0	46	1
Hosoda et al. (18)	Japan	Japan	Retrospective	Clinical isolates	Enterobacter strains	Blood agar	WGS	70	37	0	13	20	0.83
Baer et al. (19)	Israel	Israel	Retrospective	Clinical isolates	Blood\other body fluids\urine\wounds\Screening	Blood culture	PCP	48	33	0	5	10	0.9
Kanahashi et al. (4)	Japan	Japan	Prospective	Clinical isolates	–	Blood agar\MacConkey agar\Mueller-Hinton agar	PCR	53	24	0	2	27	0.96
Keshta et al. (20)	Qatar	Qatar	Prospective	Clinical isolates	–	Direct clinical blood testing	PCR	82	19	0	0	63	1
Kon et al. (21)	Israel	Israel	Retrospective	Laboratory isolate strains	Sputum\blood\urine\rectal	Mueller-Hinton agar	PCR	194	147	1	0	46	0.99
Liu et al. (22)	China	China	Prospective	Clinical isolates	Blood cultures	Blood agar	PCR	228	105	0	13	110	094
Bogaerts et al. (23)	Belgian	Belgian	Prospective	Clinical isolates	Enterobacter strains	Blood agar	PCR	161	91	0	0	70	1
Chan et al. (24)	Canada	global states	Retrospective	Global Enterobacterales isolates and clinical isolates	Enterobacter strains	\	WGS	262	207	0	5	50	0.98
Gelmez et al. (25)	Turkey	Turkey	Retrospective	Clinical isolates	*Klebsiella pneumoniae* isolates	Blood agar	PCR	224	194	0	0	30	1
Han et al. (2)	China	China	Prospective	–	Enterobacter strains	Bacterial colony	PCR	215	198	0	0	17	1
Bianco et al. (26)	Italy	Italy	Prospective	Clinical isolates	Enterobacter strains	blood agar	PCR	130	97	0	3	30	0.98
Jenkins et al. (3)	America	America	Both^1^	Clinical isolate and Laboratory isolate strains	Urine\rectal swabs/stools\blood respiratory specimens\wounds\sterile fluids\tissues	Blood agar\MacConkey agar\Mueller-Hinton agar	PCR	309	172	0	0	137	1
Potron et al. (27)	France	France	Prospective	–	–	–	PCR	168	71	0	11	86	0.93
Takissian et al. (28)	France	France	Prospective	–	–	Blood culture	PCR	205	129	0	3	73	0.99
Giordano et al. (29)	Italy	Italy	Retrospective	Clinical isolates	Clinical isolates	Blood culture direct clinical blood testing	PCR	484	233	0	4	247	0.99

^1^Prospective and retrospective; TP, true positive; TN, true negative; FP, false positive; FN, false negative.

### Methodological quality risk of bias

**FIGURE 2:**
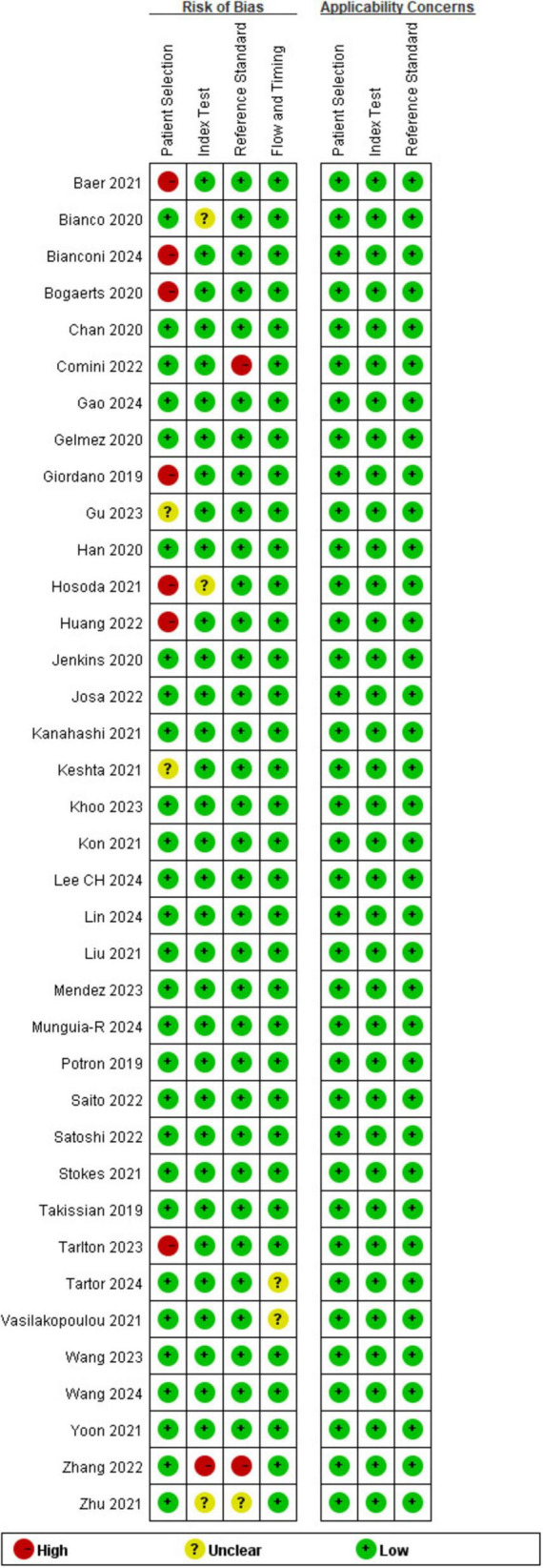
Quality evaluation of the included studies: risk of bias and applicability concerns.

The quality of individual research results was assessed, as displayed in Figure 2. We concluded that the majority of the researches had a minimal risk of bias. In the area of patient selection, approximately 19% of the researches were defined as high risk because such studies used confirmed carbapenemase-resistant bacteria for fitness-for-purpose testing and did not avoid a case-control design. In the index test domain, approximately 89% of the studies had a minimal risk of bias. The tests were carried out in compliance with the specifications of the NG-Test Carba 5 kit, and a blinded process was used to analyze the results. Some of the articles did not disclose if blinding was used and were therefore defined as having an unknown risk. Both the flow and time domains, as well as the reference standard domains, have little chance of bias.

### Publication bias

**FIGURE 3:**
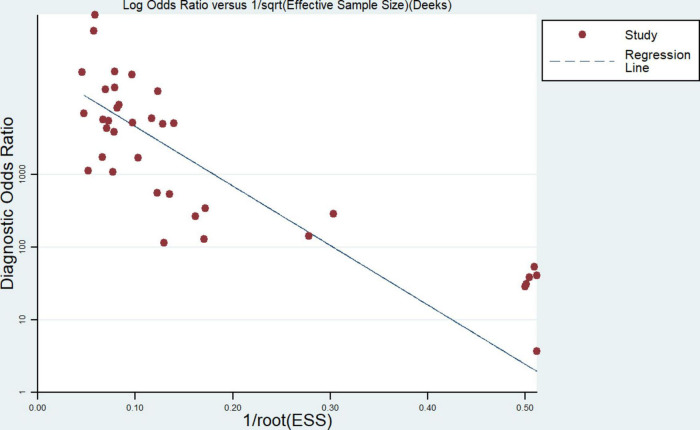
Deeks funnel plot of publications.

There was good symmetry in the Deeks funnel plot, and no evidence of publication bias was detected as we can see in Figure 3.

### Threshold effect analysis

**FIGURE 4:**
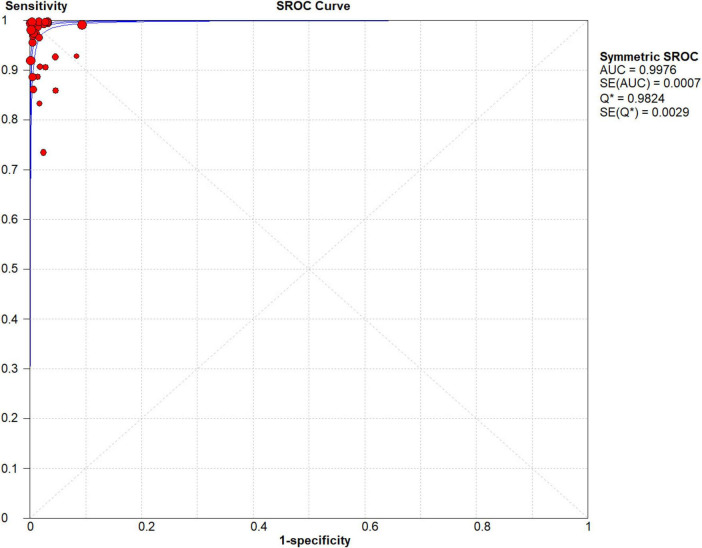
Forest plots of summary receiver operating characteristic (SROC) curve of NG-Test Carba 5 for the diagnosis of carbapenem-resistant gram-negative bacteria.

Researchers analyzed the SROC curve (Figure 4), which was not characterized by a “shoulder-arm” distribution. Consequently, we conclude that none of the included publications exhibited a threshold effect.

### SROC curves

A fixed effects model was used to fit the SROC curve. As shown in Figure 4, the AUC was 0.9976, and the Q-index was 0.9824 (SE = 0.0029). This finding suggested that the NG-Test Carba 5 has high accuracy in diagnosing carbapenemase.

### Overall meta-analysis

**FIGURE 5:**
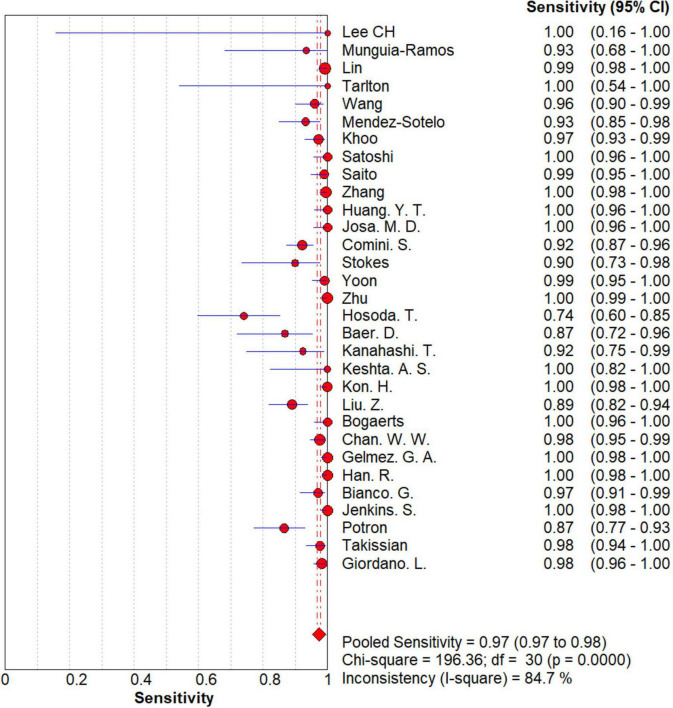
Forest plots of sensitivity of NG-Test Carba 5 for the diagnosis of carbapenem-resistant gram-negative bacteria.

**FIGURE 6:**
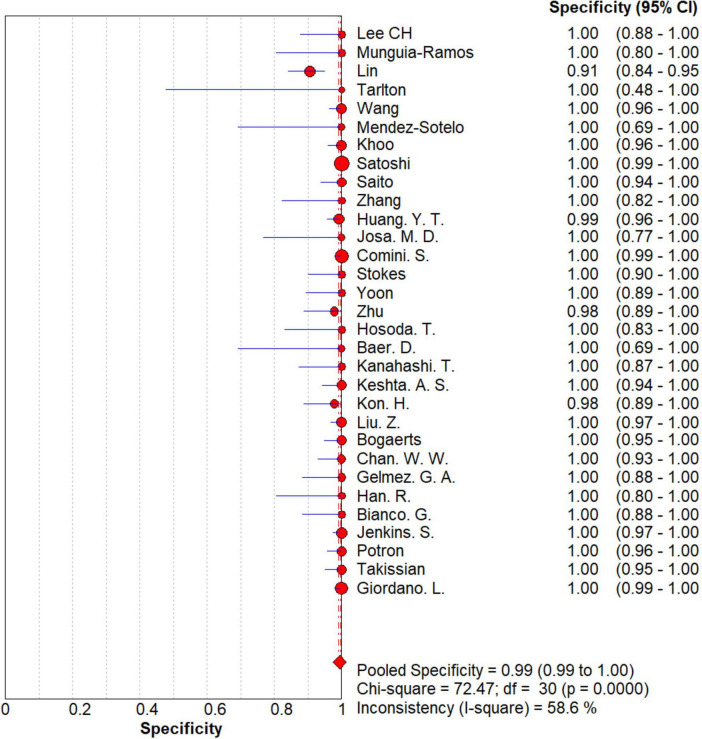
Forest plots of specificity of NG-Test Carba 5 for the diagnosis of carbapenem-resistant gram-negative bacteria.

**FIGURE 7:**
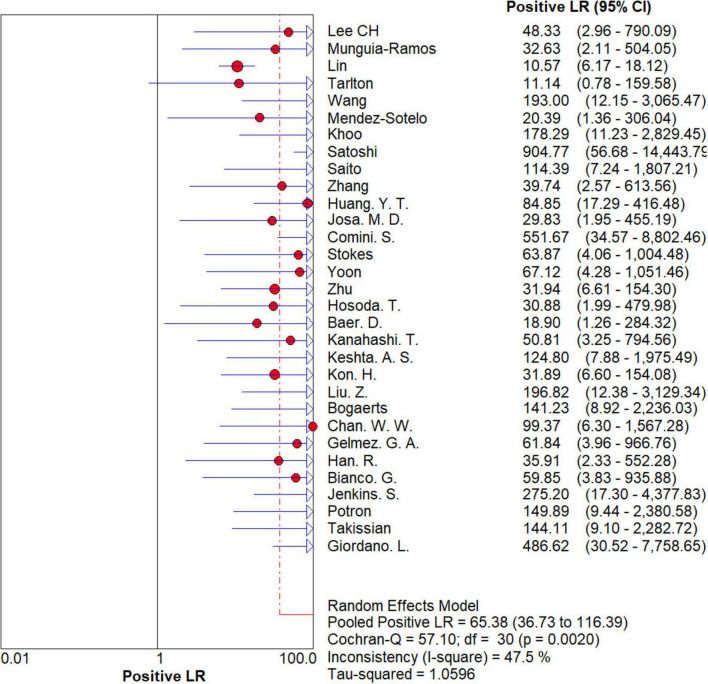
Forest plots of positive LR of NG-Test Carba 5 for the diagnosis of carbapenem-resistant gram-negative bacteria.

**FIGURE 8:**
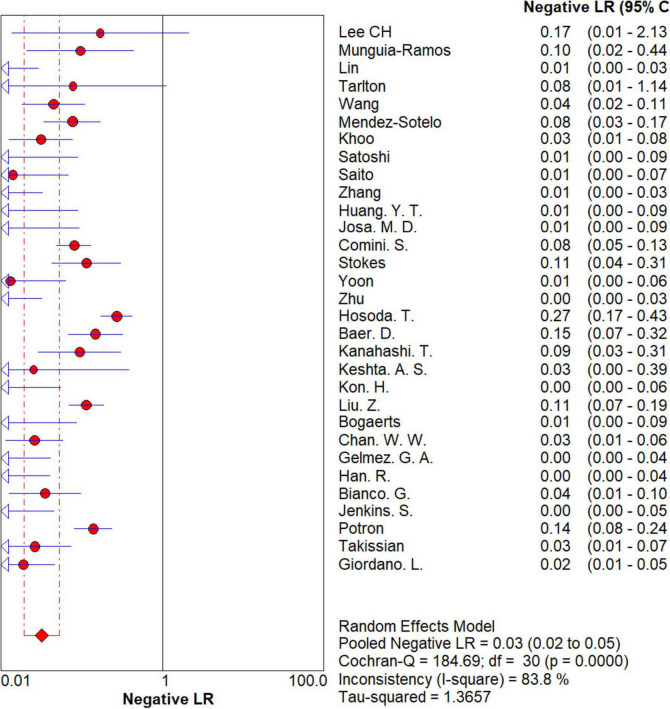
Forest plots of negative LR of NG-Test Carba 5 for the diagnosis of carbapenem-resistant gram-negative bacteria.

**FIGURE 9:**
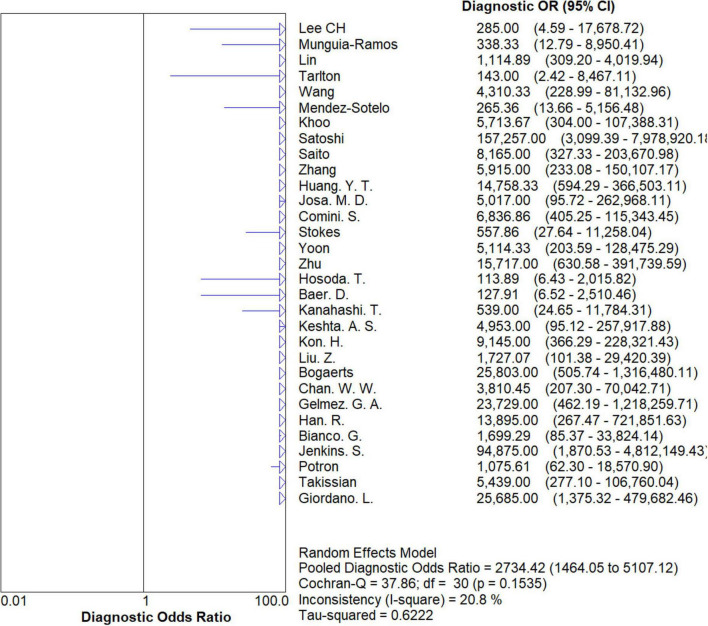
Forest plots of diagnostic OR of NG-Test Carba 5 for the diagnosis of carbapenem-resistant gram-negative bacteria.

The results are shown in Figures 5, 6. The sensitivity, specificity of NG-Test Carba 5 for diagnosing carbapenemase-type KPC, NDM, VIM, IMP, and OXA-48-like by immunochromatographic assay were 0.97 [95% CI (0.97, 0.98)], 0.99 [95% CI (0.99, 1.00)]. Additionally, the PLR and NLR were 65.38 [95% CI (36.73, 116.39)] and 0.03 [95% CI (0.02, 0.05)], respectively, and the DOR was 2,734.42 [95% CI (1,464.05, 5,107.12)]. “See Figures 7, 8, 9.” In the researches we cited, Hosoda’s study (18) tested a total of 51 strains, 48 of which were IMP-producing CPE isolates. This result may be caused by NG-Test Carba 5 giving false negatives, which explains the source of heterogeneity in the sensitivity of IMP test results in this study.

### Subgroup meta-analysis

We divided these studies into three subgroups categorized by detection outcome (Group A), bacterial species (Group B), and bacterial isolation method (Group C). Within the detection outcome group, the studies were categorized by carbapenemase type as IMP, KPC, NDM, VIM, and OXA-48, and within the bacterial species group as *Escherichia coli*, *Pseudomonas aeruginosa*, *Klebsiella pneumoniae*, and *Enterobacter cloacae*. The bacterial isolation methods categorized within the groups were blood culture, blood agar culture, direct clinical blood test, MacConkey agar culture, and Mueller-Hinton agar culture. The results are shown in Table 2.

**TABLE 2:** Subgroup analysis results.

Subgroup analysis	Sensitivity (95% CI)	Specificity (95% CI)	PLR (95% CI)	NLR (95% CI)	DOR (95% CI)	SROC	
						**AUC**	Q*
**Group A**							
IMP	0.91 (0.87–0.93)	1.00 (1.00–1.00)	181.85 (110.36–299.67)	0.12 (0.07–0.21)	1916.92 (855.57–4294.91)	0.9988	0.9898
KPC	0.99 (0.98–0.99)	1.00 (1.00–1.00)	135.44 (81.23–225.84)	0.03 (0.02–0.05)	6367.88 (3124.14–12979.55)	0.9988	0.9897
NDM	0.98 (0.97–0.99)	1.00 (1.00–1.00)	143.85 (92.86–222.83)	0.05 (0.03–0.08)	4157.72 (2137.21–8088.44)	0.9987	0.9887
VIM	0.96 (0.93–0.98)	1.00 (1.00–1.00)	176.76 (90.24–346.21)	0.11 (0.07–0.16)	2238.12 (1084.36–4619.45)	0.9929	0.966
OXA–48	0.99 (0.98–1.00)	1.00 (1.00–1.00)	171.96 (104.46–283.09)	0.05 (0.03–0.10)	4240.72 (1967.16–9141.96)	0.9989	0.9902
**Group B**							
*Escherichia coli*	0.98 (0.96–0.99)	1.00 (0.98–1.00)	15.01 (7.37–30.58)	0.06 (0.03–0.14)	342.34 (128.06–915.18)	0.9859	0.9491
*Pseudomonas* spp.	0.94 (0.85–0.98)	1.00 (0.96–1.00)	12.65 (4.30–37.22)	0.13 (0.05–0.33)	135.67 (27.59–667.17)	0.9689	0.918
*Klebsiella pneumoniae*	0.97 (0.96–0.98)	1.00 (0.99–1.00)	26.13 (11.66–58.55)	0.04 (0.02–0.09)	900.82 (262.48–3091.57)	0.9935	0.9679
*Enterobacter cloacae*	0.97 (0.90–0.99)	1.00 (0.94–1.00)	21.33 (6.37–71.40)	0.08 (0.03–0.17)	375.56 (74.07–1904.31)	0.9865	0.9503
**Group C**							
Blood culture	0.96 (0.94–0.98)	1.00 (0.98–1.00)	54.98 (19.50–154.98)	0.06 (0.03–0.12)	1045.55 (292.79–3733.65)	0.9965	0.978
Blood agar culture	0.96 (0.94–0.97)	1.00 (0.99–1.00)	72.16 (30.22–172.31)	0.03 (0.01–0.12)	2476.75 (588.68–10420.37)	0.9982	0.9857
Clinical blood direct test	0.95 (0.92–0.97)	1.00 (0.99–1.00)	291.84 (59.08–1441.61)	0.04 (0.01–0.14)	9132.32 (1458.26–57190.82)	0.999	0.9909
MacConkey agar culture	1.00 (0.98–1.00)	0.99 (0.93–1.00)	19.45 (6.49–58.30)	0.01 (0.00–0.04)	2359.86 (398.70–13967.76)	0.9976	0.9825
Mueller-Hinton agar culture	0.98 (0.96–1.00)	0.99 (0.92–1.00)	45.11 (9.26–219.64)	0.02 (0.01–0.05)	2344.62 (362.22–15176.6)	–	–

Q is the mean of Q* index. PLR, positive likelihood ratio; NLR, negative likelihood ratio; DOR, diagnostic odds ratio; AUC, area under the curve.

Group A: The pooled sensitivity was 0.91 (0.87–0.93) when the test outcome was IMP, 0.99 (0.98–0.99) when the test outcome was KPC, 0.98 (0.97–0.99) when the test outcome was NDM, 0.96 (0.93–0.98) when the test outcome was VIM, and 0.99 (0.98–1.00) when the detection outcome was OXA-48.

For Group B, when the bacterial species was *E. coli*, the total sensitivity was 0.98 (0.96–0.99), and the total specificity was 1.00 (0.98–1.00); for *Pseudomonas aeruginosa*, the total sensitivity was 0.94 (0.85–0.98), and the total specificity was 1.00 (0.96–1.00); for *Klebsiella pneumoniae*, the total sensitivity was 0.97 (0.96–0.98), and the total specificity was 1.00 (0.99–1.00); and for *Enterobacter cloacae*, the total sensitivity was 0.97 (0.90-0.99), and the total specificity was 1.00 (0.94–1.00).

In Group C, when the method of bacterial isolation was blood culture, the combined sensitivity was 0.96 (0.94–0.98), and the combined specificity was 1.00 (0.99–1.00); for blood agar culture, the combined sensitivity was 0.96 (0.94–0.97), and the combined specificity was 1.00 (0.99–1.00); for direct clinical blood test, the combined sensitivity was 0.95 (0.92–0.97), and the combined specificity was 1.00 (0.99–1.00); for MacConkey agar culture, the combined sensitivity was 1.00 (0.98–1.00), and the combined specificity was 0.99 (0.93–1.00); and for Mueller-Hinton agar culture, the combined sensitivity was 0.98 (0.96–1.00), and the combined specificity was 0.99 (0.92–1.00).

## Discussion

Currently, because there are few effective antibiotics available and because of the significant rates of morbidity and mortality, infections caused by CR-GNB are becoming more severe and are posing a serious threat to human health. According to one study (39), Asia and Africa are the two regions in the globe where carbapenem-resistant Enterobacteriaceae are most prevalent. Early diagnosis and intervention of CR-GNB can significantly reduce mortality as well as economic burden. As of this study, there is no meta-analysis evaluating evidence-based medical evidence, such as the sensitivity and specificity of the NG-Test Carba 5 for the clinical detection of CR-GNB. Therefore, this meta-analysis focused on the accuracy of the NG-Test Carba 5 for the rapid clinical identification of CR-GNB, aiming to provide strong evidence for early clinical diagnosis, infection control and mortality reduction.

In this study, researchers obtained data from 9,153 samples from 37 articles. The diagnosis of carbapenemase-type KPC, NDM, VIM, IMP, and OXA-48-like strains was performed by the immunochromatographic method NG-Test Carba 5 using the gold standard for PCR. Researchers found that the pooled sensitivity, specificity, PLR, NLR, and DOR for the NG-Test Carba 5 method were 0.97 [95% CI (0.97, 0.98)], 0.99 [95% CI (0.99, 1.00)], 65.38 [95% CI (36.73, 116.39)], 0.03 [95% CI (0.02, 0.05)], and 2734.42 [95% CI (1,464.05, 5,107.12], respectively. This indicates that the NG-Test Carba 5 has a high degree of sensitivity and specificity. Based on this gold standard, the area under the SROC curve, i.e., the AUC, was 0.9976, which is close to 1, and the Q-index was 0.9824 (SE = 0.0029), suggesting that the NG-Test Carba 5 has high accuracy in diagnosing carbapenemase typing. This is consistent with the findings of Saito et al. (5), Mendez-Sotelo et al. (8), Khoo et al. (6), Josa et al. (7), Hopkins et al. (40). Based on the analysis of the above data, it can be concluded that the NG-Test Carba 5 may be a reliable method for detecting carbapenemases regardless of enzyme typing or bacterial genus.

Our additional analysis of the publication bias of the included papers revealed no publication bias or strong symmetry in the Deeks funnel plot. In addition, the SROC curve was not characterized by a “shoulder-arm” distribution, demonstrating the absence of a threshold effect in the included publications. Researchers then performed meta-regression analyses for investigator, country, nucleic acid extraction technique, and assay using Meta-DiSk 1.4 software, which showed all *P* > 0.05, suggesting that these factors were unable to account for the heterogeneity across the included studies. Additional unidentified factors contributed to the heterogeneity among studies.

The highlight of the present study, compared to recent publications, is that a multigroup subgroup analysis was performed. Group A was categorized into five main groups according to the assay endpoints. From the study data, it was observed that the detection rate of NG-Test Carba 5 for the five carbapenemases was generally high (0.90–1.00), which is in line with previous reports (10–12). Nevertheless, when the detection endpoint was IMP, the sensitivity was significantly lower than that of the other detection endpoints (e.g., KPC, NDM, and OXA-48) for reasons that are not known to us but may be related to several factors. First, the design of the NG-Test Carba 5 assay kit manufacturer did not target a certain IMP phenotype, which led to a lower detection rate (14). Second, the possible presence of cross-reactive proteins in *Acinetobacter baumannii* (6) as well as the loss of CMY-2 AmpC β-lactamase and the loss of pore proteins (OmpF/OmpC) in Enterobacteriaceae spp. (12) may cause false-negative or false-positive results. Third, the diversity of gene sequences within the IMP family, mutability (40), and low expression (9, 11) may be the cause of assay failure. In the aforementioned studies, the NG-Test Carba 5 yielded failed test outcomes for some IMP types, such as IMP-8, IMP-13, IMP-14, and so on. This finding is the same as the statistical results we obtained for evidence-based medicine.

Notably, the results of the NG-Test Carba 5 assay for IMP enzymes revealed many inconsistencies. Some of the IMPs detected in the studies of Potron et al. (27) and Hopkins et al. (40) were false-negative, whereas Khoo described multiple false-positive assays for IMP in *Acinetobacter baumannii* in his study (6). Since it was not possible to determine the root cause of this result, more isolates from different regions are needed to validation. For false-positive results, Tarlton et al. (30) found that overloading of the LFA resulted in false positives and that instructions should be followed, and suggested that the developers of the NG-Test Carba 5 could have added inoculum schematics to circumvent the occurrence of false-positive results. Interestingly, in terms of false-negative results, one study (19) revealed the relationship between the bacterial concentration in blood cultures and assay sensitivity. For the NG-Test Carba 5 to recognize carbapenemase, there is a certain minimum concentration limit, below which the bacterial concentration may lead to negative test results with reduced sensitivity. This factor may have led to a certain amount of heterogeneity, but few articles have verified this idea.

In group B, this study focused on the differences in sensitivity and specificity between different bacterial species. The results showed that the detection outcomes for *E. coli*, *Klebsiella pneumoniae* and *Enterobacter cloacae* did not differ significantly, with NG-Test Carba 5 showing high sensitivity for *Enterobacter cloacae* despite the low number of previous studies on this bacterium alone. In contrast, the poor identification efficiency of NG-Test Carba 5 against carbapenemase-producing *Pseudomonas aeruginosa* is consistent with what was described by Mendez-Sotelo et al. (8), and the prevalent environment in which the bacteria are found may contribute to this result. However, Potron et al. (27) recommended the use of an upcoming version of the NG-Test Carba 5, which will be launched in 2019 in countries where carbapenemase-positive *Pseudomonas aeruginosa* strains are prevalent, which would help to improve the degree of accuracy of IMP detection. As mentioned in the study, the carbapenemase types of *P. aeruginosa* are predominantly IMP and VIM, and their resistance is associated with defects in their pore proteins (OprD), which are non-transferable mechanisms located on the plasmid.

In addition, Potron et al. (41) reported that *Pseudomonas aeruginosa* also produces SPM and GIM types of β-lactamases, but these rare enzymes have not been tested by the NG-Test Carba 5. Therefore, as described in the IMP detection results above, *Pseudomonas aeruginosa* with a predominance of the IMP type can cause poor detection outcomes of the NG-Test Carba 5, and some of the specific types of enzymes are not detected, leading to false-negative results. However, it is worth noting that in the research by Volland et al. (42), it was mentioned that NG-Test Carba 5v2 is an important advancement over the original version because it can detect all different kinds of IMP without affecting its identification of the other four carbapenemases and is particularly suitable for areas where carbapenemase-producing *Pseudomonas aeruginosa* is prevalent, a conclusion that is consistent with the findings of this conclusion and is consistent with the forthcoming version of NG-Test Carba 5 mentioned by Potron et al. (27).

New versions of the NG-Test Carba 5 continue to be introduced in several types of studies to provide new solutions. Tarlton et al. (30) argue that it is necessary to compare the results of carbapenemase-specific assays with the phenotypic AST profiles of the isolates and the local epidemiology, and that if these are inconsistent, additional testing is required. This also leads to the potential impact of geographic variation in CR-GNB prevalence on instrument performance. When researchers are familiar with the local phenotyping of carbapenemase resistance assays, and when the NG-Test Carba 5 gives results that are unprecedented in local epidemiologic phenotypes, we should be more sensitive to the limitations of the assay and ask questions, which could point to certain directions in future instrumentation research. This points to a certain direction for future instrumental research.

Group C of this study, which is rarely synthesized and analyzed in current publications, was targeted at bacterial isolation methods. From the isolation culture methods included in the literature, researchers categorized five types: blood culture, blood agar culture, direct clinical blood test, MacConkey agar culture and Mueller-Hinton agar culture. The results showed that the sensitivities were all in the range of 0.95–1.00, and the specificities were all in the range of 0.99–1.00, which suggested that the NG-Test Carba 5 performed better in terms of detection across all isolation techniques. In our comparison, we found that the sensitivity of 0.95 for the direct detection of clinical blood was lower than that of other bacterial isolation methods, possibly because the NG-Test Carba 5 was not originally designed for the direct detection of clinical samples (23). Nevertheless, Nishida et al. (10) reported that direct testing of clinical samples can be used as a point-of-care test (POCT) for the rapid detection of CR-GNB. Moreover, a study (15) has shown that direct testing of clinical samples, such as rectal swabs or even fecal samples, can yield more rapid and effective results for the rapid detection of gastrointestinal colonization by the majority of gram-negative bacteria that produce carbapenemase. It is significant to note that the statistics on the Mueller-Hinton agar culture method used in this study are limited and insufficient to calculate its SROC value, and more data are needed to validate this method.

In today’s prevalent assays, Xpert Carba-R is an impressive player. However, Tarlton et al. (30) found that IMP-27 is a known test limitation of Xpert Carba-R and can be detected by NG-Test Carba 5. Moreover, Kanahashi et al. (4) found that the sensitivity of the assay for Xpert Carba-R under the same metrics was 53.8%, and the sensitivity of NG-Test- Xpert Carba-R is based on an automated real-time PCR platform, which is relatively complex and requires specialized personnel to operate, but NG-Test Carba 5 is easy to operate, without complex equipment, suitable for rapid screening, which takes less time and is suitable for initial diagnosis. Based on the potentially better performance and more compatible with the screening requirements, NG-Test Carba 5 shows a more fascinating charm.

There are several limitations to this study. First, the assay endpoints in this study did not cover subgroup analyses of complex carbapenemases or the OXA-48 family, which could result in altered performance of the NG-Test Carba 5 assay. Second, the subgroup analysis included only two data sets from the Group C Mueller-Hinton agar culture method. Therefore, we are unable to determine with certainty how sensitive the NG-Test Carba 5 is to this assay, and more studies applying this method are needed to provide reliable data to validate the present conclusions. Finally, the limitations of the NG-Test Carba 5 assay equipment led to misinterpretation of some specific carbapenemases, such as GES and IMI, which may have caused heterogeneity in the included literature.

In summary, the findings of the above meta-analysis showed that the NG-Test Carba 5 can quickly, easily, and accurately detect carbapenemase, and its high sensitivity and specificity provide novel approaches for clinical diagnosis and infection control, especially in severe environments where carbapenem-resistant bacterial infections are becoming increasingly serious in China and effective antibiotic types are scarce. However, a larger sample size and additional field methods are still needed to confirm the above conclusions.

## Data Availability

The raw data supporting the conclusions of this article will be made available by the authors, without undue reservation.
